# Prescribing Antibiotics for Children with Acute Conditions in Public Primary Care Clinics in Singapore: A Retrospective Cohort Database Study

**DOI:** 10.3390/antibiotics13080695

**Published:** 2024-07-25

**Authors:** Vivien Min Er Lee, Si Hui Low, Sky Wei Chee Koh, Anna Szuecs, Victor Weng Keong Loh, Meena Sundram, José M. Valderas, Li Yang Hsu

**Affiliations:** 1 Division of Family Medicine, Department of Medicine, Yong Loo Lin School of Medicine, National University of Singapore, Singapore 119228, Singapore; sky_wc_koh@nuhs.edu.sg (S.W.C.K.); anna.szuecs@nus.edu.sg (A.S.); victorloh@nus.edu.sg (V.W.K.L.); jmvalderas@nus.edu.sg (J.M.V.); 2 Department of Family Medicine, National University Health System, 1E Kent Ridge Rd, Singapore 119228, Singapore; 3 National University Polyclinics, National University Health System, Singapore 609606, Singapore; si_hui_low@nuhs.edu.sg (S.H.L.); meena_sundram@nuhs.edu.sg (M.S.); 4 Saw Swee Hock School of Public Health, National University of Singapore, Singapore 117549, Singapore; mdchly@nus.edu.sg

**Keywords:** paediatric, primary care, antimicrobial use

## Abstract

Data on primary care antibiotic prescription practices for children in Singapore, which are essential for health care policy, are lacking. We aimed to address this gap and to benchmark prescription practices against international standards. A retrospective cohort database study on antibiotic prescriptions for children (aged < 18 years) who visited six public primary care clinics in Singapore between 2018 and 2021 was conducted. Data were categorised according to the World Health Organization’s Access, Watch, Reserve (WHO AWaRe) classification. Quality indicators from the European Surveillance of Antimicrobial Consumption Network (ESAC-Net) and the National Institute for Health and Care Excellence (NICE) guidelines were used as a measure of appropriateness of antibiotic prescribing at the individual and overall patient level. In 831,669 polyclinic visits by children between 2018 and 2021, there was a significant reduction in mean antibiotics prescribed per month during pandemic years (2020–2021) compared to pre-pandemic (2018–2019) (MD 458.3, 95% CI 365.9–550.7). Most prescriptions (95.8%) for acute conditions fell within the WHO AWaRe “Access” group. Antibiotic prescription significantly exceeded (55.2%) the relevant quality indicator for otitis media (0–20%). The proportion of children receiving appropriate antibiotics for acute respiratory infections (*n* = 4506, 51.3%) and otitis media (*n* = 174, 49.4%) was low compared to the quality indicator (80–100%). There is a need to develop local evidence-based primary care antibiotic guidelines, as well as to support the development of stewardship programmes.

## 1. Introduction

Antimicrobial resistance (AMR) is a global public health threat that is exacerbated by the misuse and overuse of antimicrobial agents. Drug-resistant infections are more difficult and costly to treat, resulting in increased mortality and higher socioeconomic impact [1,2]. Efforts to decrease antibiotics’ inappropriate use can reduce potential drug reactions and slow down the development of AMR [3].

Most antibiotics are prescribed in primary care, often inappropriately for acute viral respiratory infections, where they are not indicated [4]. Studies have shown that an average of a third of primary care paediatric consultations for acute respiratory infections result in the prescription of antibiotics [5,6].

Multiple guidelines for the management of common paediatric infections have been published, including those by the World Health Organization (WHO) [7] and the National Institute for Health and Care Excellence (NICE) [8]. The European Surveillance of Antimicrobial Consumption Network (ESAC-Net), a Europe-wide network of national surveillance systems, provides European reference data on antimicrobial consumption in both community and hospital settings. ESAC-Net has additionally developed and validated disease-specific antibiotic prescribing quality indicators for primary care [9]. Yet Singapore lacks local antibiotic prescribing guidelines for paediatric infections and, thus, the gaps in recent practices remain unclear [10]. No previous study has ever estimated the prevalence and nature of primary care antibiotic prescription for children in Singapore.

In Singapore, primary care is provided through an island-wide network of outpatient polyclinics and clinics run by private general practitioners (GPs). There are currently 23 polyclinics and about 1800 GP clinics [11]. Primary care practitioners see community outpatients of all ages, including newborns and children, with conditions ranging from acute to chronic [12]. On average, about 6 million people attend the polyclinics annually [13].

During the COVID-19 pandemic, changes in patients’ behaviour in terms of seeking health care and physicians’ prescription patterns may have affected community antibiotic prescriptions [14]. A local study performed in outpatient setting showed a reduction rate in antimicrobial prescriptions for adults in 2020 compared to before the pandemic [15]. However, there were no local studies conducted in the paediatric population.

The lack of essential information impedes the development of local paediatric antibiotic guidelines for primary care and is a barrier for antimicrobial stewardship in the post-pandemic era.

We, therefore, aim to describe and assess the quality of antibiotic prescribing practices for acute paediatric infections in Singapore’s public primary care settings. Because of the potential impact of the COVID-19 pandemic on prescribing behaviours, we also aim to establish if it has resulted in different prescribing patterns.

## 2. Materials and Methods

### 2.1. Study Design and Setting

We conducted a retrospective cohort database study in six polyclinics from the western primary care cluster of Singapore: National University Polyclinics. The cluster comprises seven outpatient clinics [16], although the most recent only opened in 2020, and was therefore not included in the study, which spanned from 1 January 2018 to 31 December 2021.

### 2.2. Participants

The subjects were outpatients <18 years of age who were prescribed oral or topical antibiotics while attending the polyclinics. Patients who were prescribed antibiotics for prophylaxis or received treatment for more than 14 days were excluded, as the study focused on acute conditions.

### 2.3. Data Sources and Variables

Demographic and clinical data, along with prescriber information, were extracted from the electronic health records. Data were then cleaned using R (Version 4.2.0) to standardise variables and remove irrelevant and incomplete data. De-identification was performed by a centralised, trusted third party (an institutional research office) before analysis by the study team. One clinic only had data available from 2021, as it had used a different electronic health record system prior to 2021.

Institutional-level data on the total number of visits for acute respiratory infections (ARI) and otitis media were collected for comparison against EASC-Net quality indicators. The antibiotic prescription rate was derived by dividing the number of prescriptions over the total number of patient visits. For the purposes of this study, each antibiotic prescribed equates to one antibiotic prescription, regardless of the number of visits by the same patient.

Visit diagnoses were coded using the International Classification of Disease (ICD-10) [17]. To analyse antibiotic prescriptions by diagnoses, visits including a prescription of oral antibiotics were grouped into categories based on the indicated diagnosis: respiratory, skin, genitourinary, gastrointestinal, infectious disease, and dental conditions. Prescriptions for which indications could not be ascertained were listed as ‘undefined’. The diagnosis categorisation was conducted independently by two trained family physicians based on the ICD-10 [17], and any discrepancies were de-conflicted.

Antibiotic classification followed the 2021 WHO AWaRe classification [18], a tool used to rationalise antibiotic use. AWaRE stands for Access, Watch, Reserve. It groups antibiotics into three main categories based on their strength and potential impact on AMR. ‘Access’ antibiotics are first- or second-line treatments for common infections that should be widely accessible, while ‘Watch’ antibiotics should be restricted to a limited group of well-defined syndromes, and ‘Reserve’ antibiotics should be only applied as a last resort to treat multi- or extensively drug-resistant bacteria.

When antibiotics were prescribed for visits with multiple diagnoses, we used a tiered ranking logic system to select infective conditions over non-infective conditions, and prioritised the ranking of conditions in terms of which antibiotics were often required, until each antibiotic prescription belonged to only one category. Prescriptions for which indications remained uncertain were grouped under ‘multiple diagnoses’. All antibiotics prescribed by dentists were assumed to be for dental conditions.

To ensure data validity and accuracy of the above ranking classification, 100 records were randomly selected and audited by hand. In all cases, the audit verified the ranking classification decisions.

### 2.4. Quality Standards Assessment

As Singapore has no current national primary care guidelines on antibiotic usage for its paediatric population, we estimated the appropriateness of antibiotic use for common conditions based on the NICE guidance [8] (Table 1). The overall quality of primary care antibiotic prescriptions was benchmarked against the ESAC-Net quality indicators [9] for the three conditions where indicators were applicable to children: acute respiratory infection (ARI), acute tonsillitis, and acute otitis media (Table 2).

### 2.5. Statistical Methods

R (Version 4.2.0), IBM SPSS Statistics Version 29.0, and Microsoft Excel 2010 were used in data analysis.

An independent samples *t*-test [19] was used to detect mean difference (MD) in the prescription rates before and during the COVID-19 pandemic.

### 2.6. Ethics

The study was approved by the NHG Domain-Specific Review Board on 26 August 2022 (2022/00509).

## 3. Results

Out of 831,669 paediatric patient visits between 2018 and 2021, oral and topical antibiotics were prescribed for 19,325 (2.3%) and 20,692 (2.5%) visits, respectively (Table 3 and Table 4). There was a significant reduction in the mean antibiotic prescription counts per month between the COVID-19 pandemic years of 2020 and 2021 compared to the pre-pandemic years of 2018 and 2019 (MD 458.3, 95% CI 365.9-550.7).

**Figure 1:**
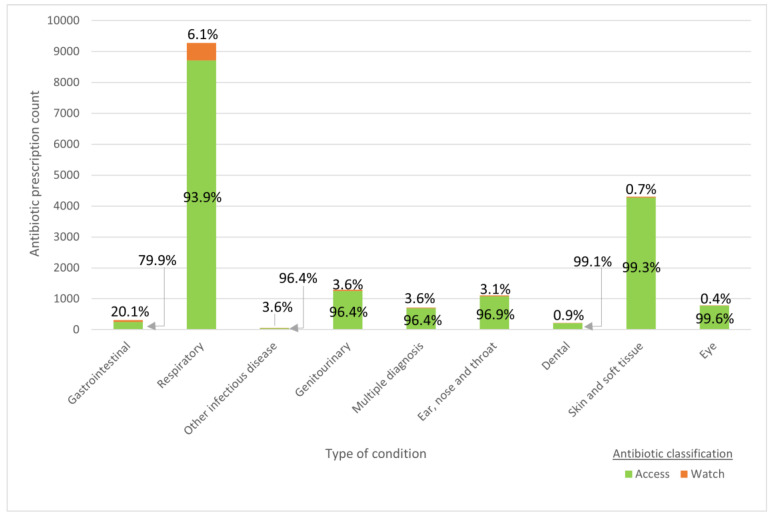
AWaRe classification of oral antibiotics prescribed by type of condition.

Most oral antibiotics were prescribed for respiratory and skin and soft tissue conditions (70.3%), although the rationale for a significant minority (>5% each year) could not be ascertained (Table 3). Only a small proportion of prescriptions (*n* = 820, 4.2%) were “Watch” group antibiotics. There were no prescriptions under the “Reserve” group antibiotics (Figure 1). The greatest number of “Watch” group antibiotics were prescribed for respiratory conditions (*n* = 562, 6.1%), with clarithromycin (*n* = 524, 5.6%) being the most prescribed. Significantly, one-fifth of antibiotics prescribed for gastrointestinal conditions were “Watch” antibiotics (*n* = 63, 20.1%), with ciprofloxacin (*n* = 48, 15.3%) being the most prescribed. The most common diagnosis in which antibiotics were prescribed for gastrointestinal conditions was gastroenteritis (52.6%).

Topical antibiotic prescriptions rate remained stable from 2018–2021 (Table 4). Most topical antibiotics were prescribed for ophthalmic and skin and soft tissue indications. 

When benchmarked against ESAC-Net quality indicators, oral antibiotic prescriptions for otitis media exceeded the acceptable range (*n* = 352, 55.2% of all otitis media visits). For acute respiratory infections (ARI), antibiotic prescriptions were within the acceptable range, with only 4.25% (*n* = 8790) of ARI visits resulting in antibiotic prescription. Only about half of antibiotic prescriptions for ARI and otitis media were recommended antibiotics by the NICE guidelines [8], below the acceptable range. The most preferred antibiotic that was not recommended for ARI (*n* = 3550, 39.6%) and otitis media (*n* = 179, 45.4%) was amoxicillin/clavulanate. The use of quinolones, which are “Watch” antibiotics, was within the acceptable range (Table 5). There were insufficient data for tonsillitis (*n* = 0), hence it was excluded.

## 4. Discussion

### 4.1. Discussion

This study is the first to document the prevalence and nature of primary care antibiotic prescription for children in Singapore, highlighting key trends and areas for improvement. Although most antibiotics prescribed were “Access” group antibiotics, which have generally less adverse drug reactions and a narrower spectrum of activity with lower potential for causing AMR, a number of opportunities for improved prescribing were identified, particularly for respiratory and gastrointestinal conditions.

The prescribing rate for oral and topical antibiotics in our study is comparable to our previous local study conducted in adults in Singapore [15]. There are different ways to represent antibiotic prescription rate but no gold standard. Other studies used the prevalence rate (the number of people who received at least one antibiotic drug prescription per 100 individuals in the population) and prescription rate (number of antibiotic drug prescriptions per person per year), whereas we have reported visit-based rates, i.e., the number of antibiotics prescribed divided by the total number of medical visits. This may explain the difference in rates between our findings and other studies [20,21]. We chose visit-based rates of antibiotic prescriptions, as this is the method followed by the United States Center for Disease Control and Prevention (CDC) [22], and also used in our previous research article on local antibiotic prescription in adults, which was the primary source of comparison for our study [15].

Similar to other European studies [23,24], antibiotic prescriptions for otitis media far exceeded the international recommendations. Guidelines recommend that watchful waiting can be applied in selected children with non-severe acute otitis media [8,25,26]. Physicians may not be updated on these guidelines or may feel more comfortable with or pressured into administering antibiotics promptly to reassure children and their parents. The rationale for prescription practices merits further study to provide a stronger evidence base, which is particularly sparse in studies in South East Asia [27].

The appropriateness of antibiotic choice for ARI and otitis media were below standard. Compared to European studies, which showed better adherence to recommended antibiotics, more local physicians inappropriately prescribed amoxicillin/clavulanate, a broad-spectrum antibiotic with increased risk of AMR [28]. This may be due to an interplay of internal and external factors, including drug familiarity, other physicians’ common practices, time pressure, previous patients’ experience, pressure from patients due to a lack of patient education, and even financial issues [29,30,31,32]. Further in-depth studies, possibly with exploratory qualitative designs, should elucidate the reasons for physicians’ antibiotic prescription preferences in local settings and identify barriers to compliance with guidelines.

One-fifth of antibiotics administered for gastrointestinal conditions, especially gastroenteritis, were “Watch” group antibiotics, mainly ciprofloxacin. Routine use of antibiotics is not recommended, as most gastroenteritis is self-limiting [33,34]. Moreover, ciprofloxacin should generally be reserved for serious conditions in paediatric populations to ensure that the benefits outweigh the risks. The United States Food and Drug Administration indeed warns against potentially severe adverse reactions, including tendinopathy and tendon rupture, peripheral neuropathy, and neurological effects [35,36,37]. This preference for ciprofloxacin could arise from recommendations for its use in adults with traveller’s diarrhoea and dysentery, and the relatively low frequency and good reversibility of the drug’s side effects [38,39,40]. However, the present study does not provide the necessary evidence to back these speculations and highlights the need for future research focusing on the reasons for this preference.

There was a significant drop in antibiotic prescriptions during the COVID-19 pandemic [15]. This could be due to public health measures, increased public awareness, and increased accessibility to COVID-19 testing. Further studies should confirm whether in the post/pandemic situation, previous patterns recur.

Similar to a previous local study with an adult population [14], a significant minority of antibiotic prescriptions with unknown diagnosis made the assessment of antibiotic indications challenging. Quality improvement projects could be helmed to improve accurate diagnosis coding, while the development of research methods is warranted to improve our attribution ability.

Our findings were limited to the western cluster of Singapore’s public primary care institutions. It remains unknown to what extent they are generalisable to the two other public primary care clusters of the country or to private primary care settings. However, given that Singapore’s primary care physicians have mostly trained locally and Singapore offers relatively uniform levels of medical training across its three medical schools, the findings likely highlight broader gaps, for instance, regarding antibiotic prescription for otitis media and choice of antibiotic for ARI, otitis media, and gastrointestinal conditions. These gaps need to be more broadly investigated in both public and private settings.

### 4.2. Limitations

This study was limited to the western cluster of Singapore’s public primary care clinics and did not include private clinics. Further studies are needed in private practices to better reflect antibiotic use and prevalence.

The study was limited to the period of 2018–2021, as this study’s design mirrored the design and timeframe of a previous study on antibiotics use in adults [15]. It furthered our understanding of how the pandemic affected prescription trends, yet its scope did not extend to post-pandemic practices at the time of its conceptualisation, which still occurred in the midst of the pandemic.

Using international guidelines to audit local prescription practices may be inappropriate due to the differences in aetiology, antimicrobial sensitivity and availability, and prescribing practices. Delayed antibiotic prescribing could also be a potential source of bias in interpreting the results; however, it is unlikely to account entirely for the high prescription rates. The authors of the ESAC-Net guidelines acknowledge that delayed prescriptions may be a source for potential variation, which may bias the interpretation of their quality indicators [24].

Visit diagnoses were grouped into major systems, as there would have been too many different diagnoses to list otherwise. The groupings are the same as those in our previous paper on the adult population [15]. Only the specific diagnoses of ARI and otitis media were described separately, as these conditions can be compared to EASC-Net quality indicators.

Missing diagnosis codes and multiple visit diagnoses might have affected the robustness of the tiered ranking system in diagnosis classification, resulting in a misrepresentation of antibiotics prescribed for certain diagnoses. Medical record reviews using machine learning could ameliorate prescription quality.

## 5. Conclusions

This study showcases the prevalence and appropriateness of antibiotic prescriptions for children within a segment of public primary care in Singapore. The gaps identified were antibiotic prescription for otitis media and choice of antibiotic for ARI, otitis media, and gastrointestinal conditions. These findings pave the way for further research to identify the reasons behind these gaps and lay the foundation for adapting antimicrobial guidelines and stewardship programmes to Singapore’s primary care setting.

## Figures and Tables

**Table 1:** Antibiotics recommended by the NICE guidelines for ARI, tonsillitis, and otitis media.

	ARI *	Acute Tonsillitis	Acute Otitis Media
First-line	Amoxicillin	Penicillin V	Amoxicillin
Second-line or Penicillin allergy	Clarithromycin		

* Acute respiratory infection.

**Table 2:** EASC-Net quality indicators.

Infection	Age Range	Acceptable Range Receiving Antibiotics, %	Acceptable Range Receiving Recommended Antibiotics, %	Acceptable Range Receiving Quinolones, %
ARI *	>1 year old	0–20	80–100	0–5
Acute tonsillitis				
Acute otitis media	>2 years old			

* Acute respiratory infection.

**Table 3:** Oral antibiotic prescriptions, 2018–2021.

Variable Prescription Rate Per Year, *n* (%)	2018	2019	2020	2021
	*n* = 6371 (2.6%)	*n* = 6798 (2.8%)	*n* = 3529 (2.0%)	*n* = 2627 (1.5%)
Final diagnosis, *n* (%)				
Dental	47 (0.7%)	61 (0.9%)	65 (1.8%)	39 (1.5%)
ENT (ear, nose, and throat)	328 (5.2%)	383 (5.6%)	239 (6.8%)	160 (6.1%)
Eye	191 (3.0%)	232 (3.4%)	190 (5.4%)	171 (6.5%)
Gastrointestinal	114 (1.8%)	120 (1.8%)	48 (1.4%)	31 (1.2%)
Genitourinary	331 (5.2%)	366 (5.4%)	280 (7.9%)	319 (12.1%)
Other infectious disease	21 (0.3%)	20 (0.3%)	9 (0.3%)	5 (0.2%)
Multiple diagnoses	257 (4.0%)	274 (4.0%)	144 (4.1%)	45 (1.7%)
Respiratory	3876 (60.8%)	3666 (53.9%)	1138 (32.3%)	598 (22.8%)
Skin and soft tissue	868 (13.6%)	1276 (18.8%)	1167 (33.1%)	991 (37.7%)
Undefined	338 (5.3%)	400 (5.9%)	249 (7.1%)	268 (10.2%)

Prescription rate per year, %.

**Table 4:** Topical antibiotic prescriptions, 2018–2021.

Variable Prescription Rate Per Year, *n* (%)	2018	2019	2020	2021
	*n* = 6278 (2.6%)	*n* = 6061 (2.5%)	*n* = 4383 (2.5%)	*n* = 3970 (2.3%)
Final diagnosis, *n* (%)				
ENT (ear, nose, and throat)	807 (12.9%)	699 (11.5%)	545 (12.4%)	433 (10.9%)
Eye	3039 (48.4%)	2913 (48.1%)	1658 (37.8%)	1443 (36.4%)
Skin and soft tissue	2432 (38.7%)	2449 (40.4%)	2180 (49.7%)	2094 (52.8%)

**Table 5:** Comparative assessment of acute respiratory conditions and otitis media in relation to EASC-Net criteria.

	Acute Respiratory Infection	Otitis Media
Receiving antibiotics, % (*n*)	4.3 (8790)	55.2 (352)
Receiving recommended antibiotics, % (*n*)	51.3 (4506)	49.4 (174)
Receiving quinolones, % (*n*)	0.1 (11)	0.3 (1)

ESAC-Net acceptable range, %, for receiving antibiotics: 0–20%. EASC-Net acceptable range, %, for receiving recommended antibiotics: 80–100%. EASC-Net acceptable range, %, for receiving quinolones: 0–5%.

## Data Availability

The data presented in the study are available on request from the corresponding author.
